# Supplementary material for: Molecular basis of maintaining an oxidizing environment under anaerobiosis by soluble fumarate reductase
Source: Nat Commun. 2018 Nov 19;9:4867. doi: 10.1038/s41467-018-07285-9 (PMC6242907; doi:10.1038/s41467-018-07285-9)
Supplement: Supplementary file 1 — Supplementary Information [file 41467_2018_7285_MOESM1_ESM.pdf]

Molecular basis of maintaining an oxidizing environment under anaerobiosis by soluble fumarate reductase

Sunghwan Kim[1,#,*,@], Chang Min Kim[2,#], Young-Jin Son[1,#], Jae Young Choi[3], Rahel K. Siegenthaler[4], Younho Lee[5], Tae-Ho Jang[1], Jaeyoung Song[1], Hara Kang[6], Chris A. Kaiser[4], and Hyun Ho Park[2,*]

[1]New Drug Development Center, Daegu-Gyeongbuk Medical Innovation Foundation, Daegu, South Korea.
[2]College of Pharmacy, Chung-Ang University, Seoul 06974, South Korea.
[3]Department of Biochemistry, Yeungnam University, Gyeongsan, South Korea.
[4]Department of Biology, Massachusetts Institute of Technology, Cambridge, MA 02139, USA.
[5]College of Pharmacy and Yonsei Institute of Pharmaceutical Sciences, Yonsei University, 85 Songdogwahak-ro, Yeonsu-gu, Incheon, Republic of Korea.
[6]Division of Life Sciences, College of Life Science and Bioengineering, Incheon National University, Incheon, Republic of Korea.
[@] Current address: R&D center, Polus Inc., 9 Songdomirae-ro, Yeonsu-gu, Incheon, South Korea.

Supplementary Figures

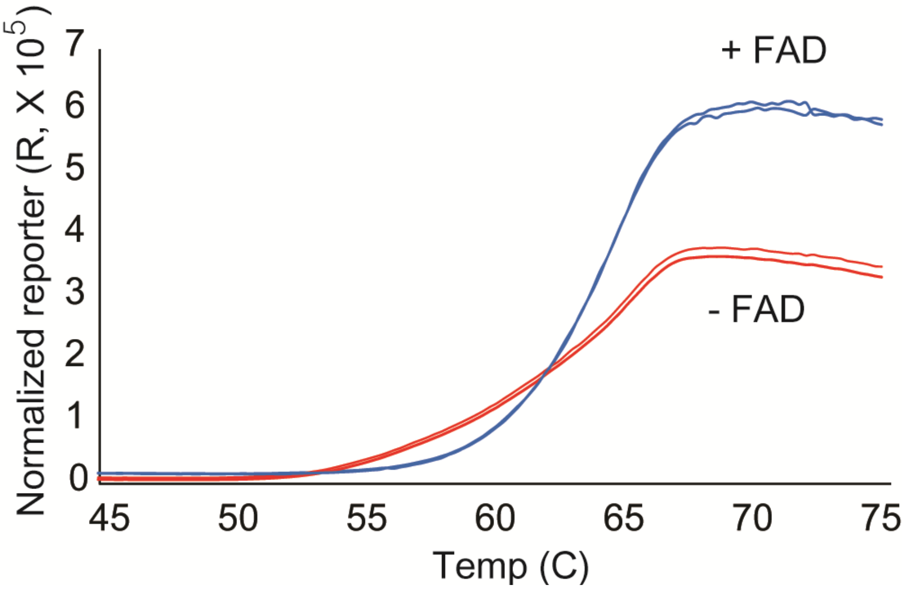

Supplementary Figure 1
Changes in thermal stability of Osm1 depending on FAD binding. Red lines indicate purified recombinant Osm1 and blue lines indicate recombinant Osm1 incubated with exogenous FAD.

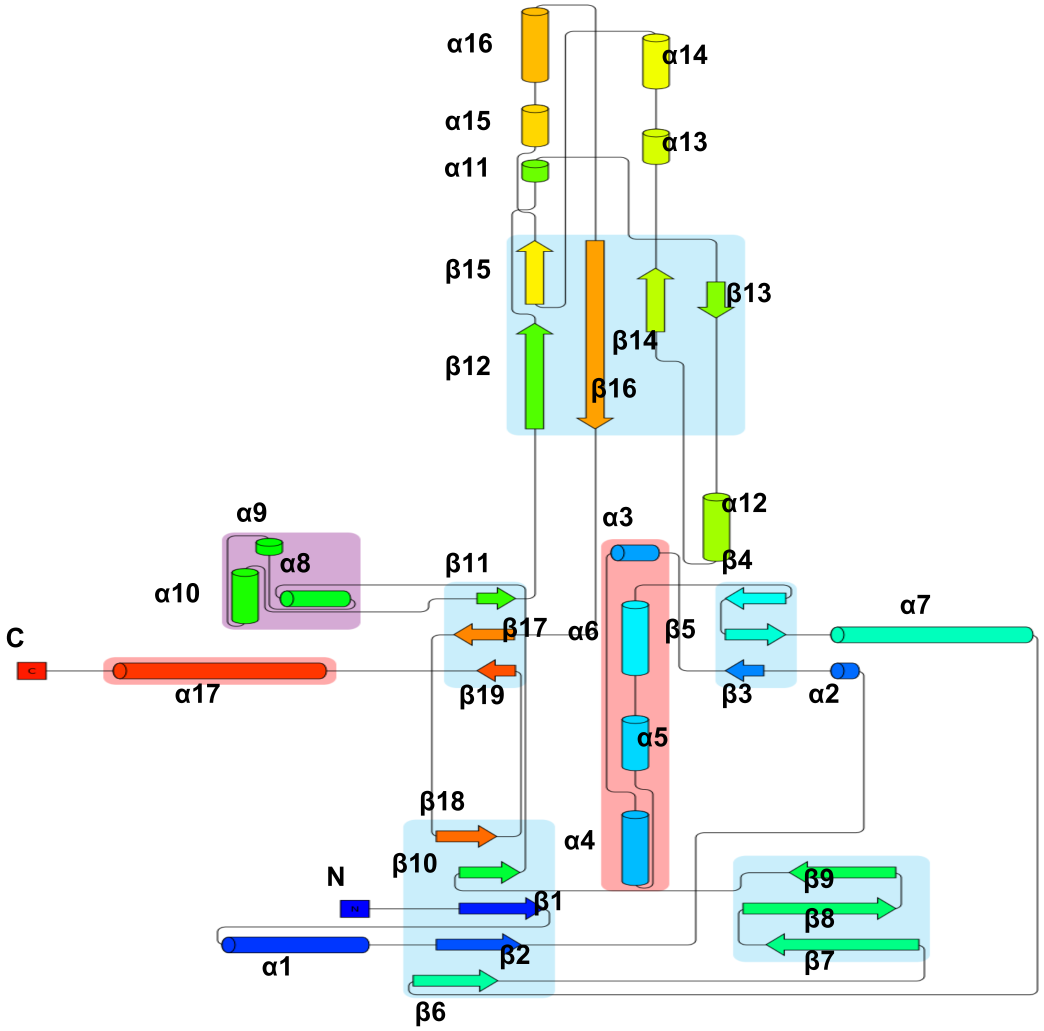

Supplementary Figure 2
Topology model of the secondary structure of Osm1. The model was constructed using Pro-origami.

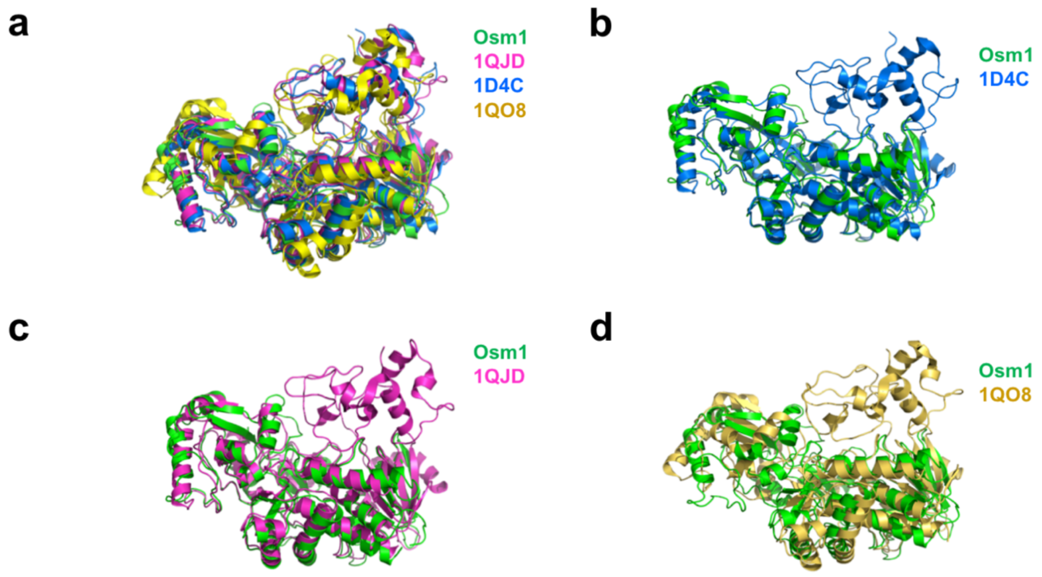

Supplementary Figure 3

Structural comparison of Osm1 with three Fcc3 family members. a Osm1 is superimposed with three known Fcc3 structures, 1QJD: Fcc3 from *Shewanella frigidimarina*, 1D4C: Fcc3 from *Shewanella putrefaciens*, 1QO8: open form of Fcc3 from *Shewanella frigidimarina.* b–d, Pairwise superposition. Osm1 is colored green and each counterpart is colored blue for 1D4C (b), magenta for 1QJD (c), and yellow for 1QO8 (d).

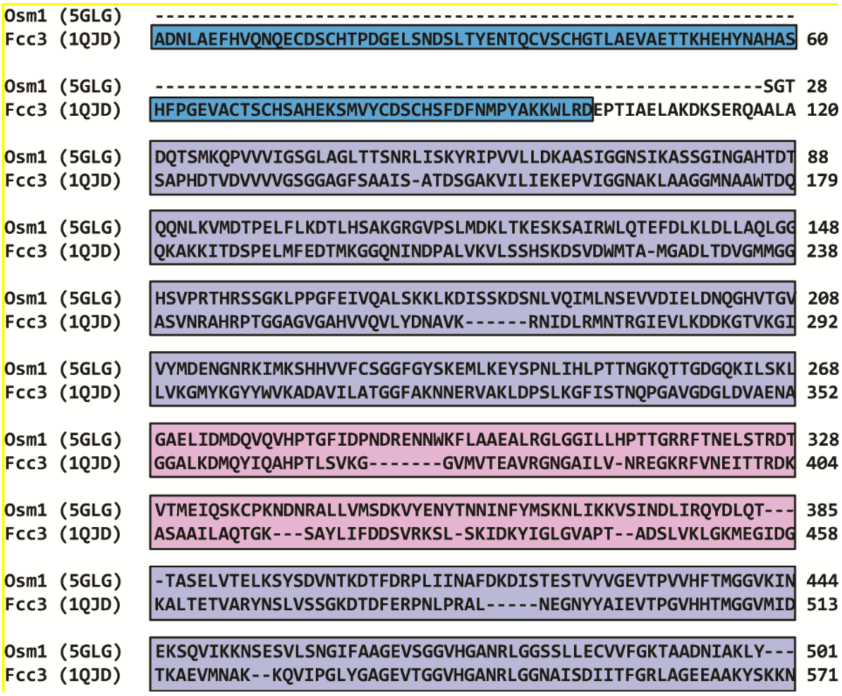

Supplementary Figure 4

Sequence alignment between Osm1 and a representative soluble fumarate reductase, Fcc3 from *Shewanella frigidimarina*. Domains are indicated by different colors. The cyan color indicates the heme domain, which is absent in Osm1. The flavin domain and the clamp domain are indicated by blue and pink colors, respectively.

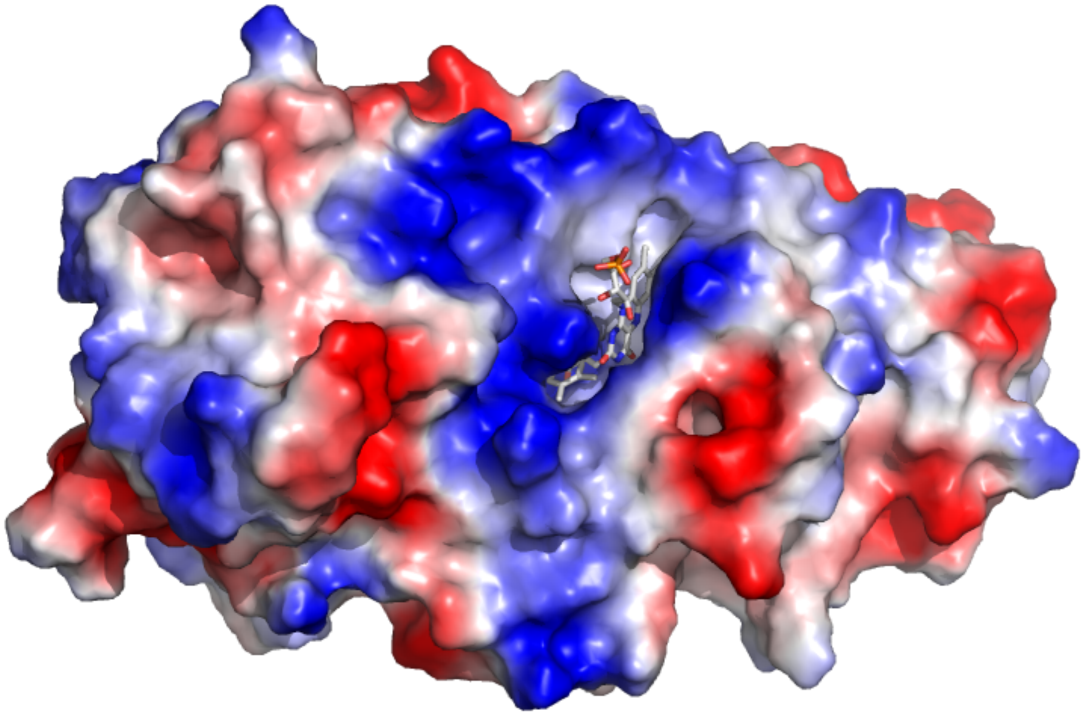

Supplementary Figure 5
Electrostatic surface presentation of the second FAD bound Osm1. The second FAD molecule in the well-defined pocket is shown as a stick model

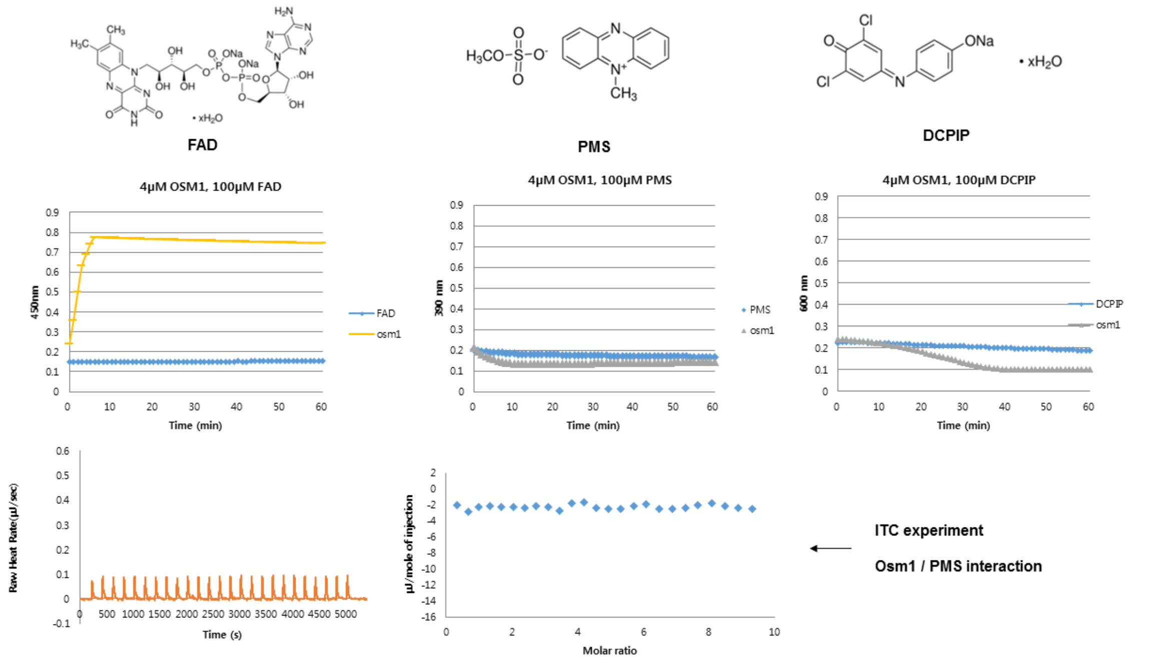

Supplementary Figure 6

Binding analysis of non-flavin electron carrier molecules to Osm1. Anaerobic oxidation of electron carrier molecules, phenazine methosulfate (PMS) and 2,6-dichlorophenolindophenol (DCPIP) by Osm1 and fumarate have been compared to that of FAD. All electron carrier molecules, 100 uM, were reduced by sodium dithionite, then incubated with 4 uM Osm1 and 1mM under air-tight sealed plates. Oxidation process was monitored over time at appropriate wavelengths specific to each molecules. In contrast to FAD, neither PMS or DCPIP was re-oxidized by Osm1. Additionally, PMS did not show binding to Osm1 in isothermal titration calorimetry experiment, indicating the second FAD binding site is quite specific to flavin molecules.

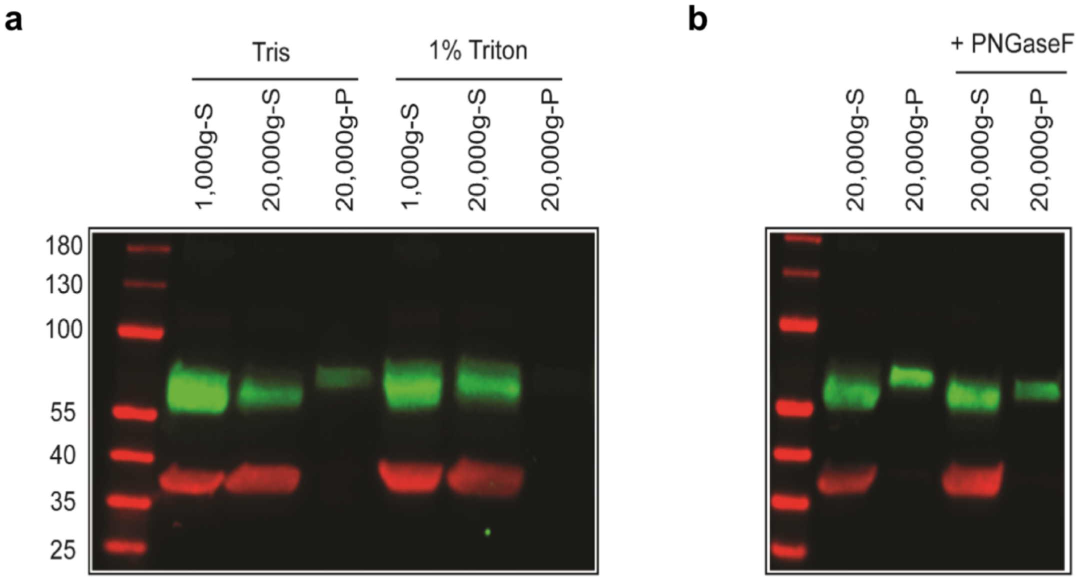

Supplementary Figure 7
Fractionation of ER- and mitochondria-resident Osm1. a Cell lysates from *S. cerevisiae* expressing HA-tagged Osm1 were fractionated into the soluble fraction (20,0000g-S) and the insoluble fraction (20,000-P) by centrifugation at 20,000 *g* in 50 mM Tris, 150 mM NaCl, and protease inhibitors, with or without 1% Triton-X100. Osm1 in the insoluble fraction without Triton-X100 appeared to be slightly larger than that in the soluble fraction (20,000g-S). In the presence of 1% Triton-X100, Osm1 was not found in the insoluble fraction, indicating that Osm1 is associated with the membrane. Osm1 (green) and GAPDH (red) were immunoblotted using anti-HA and anti-GAPDH antibodies and visualized with the appropriate IR-conjugated secondary antibodies using Odyssey (Li-Cor). b Osm1 in the insoluble fraction without Triton-X100 was sensitive to the deglycosylase, PNGaseF (NEB), suggesting that membrane-bound Osm1 is an ER-resident isoform.

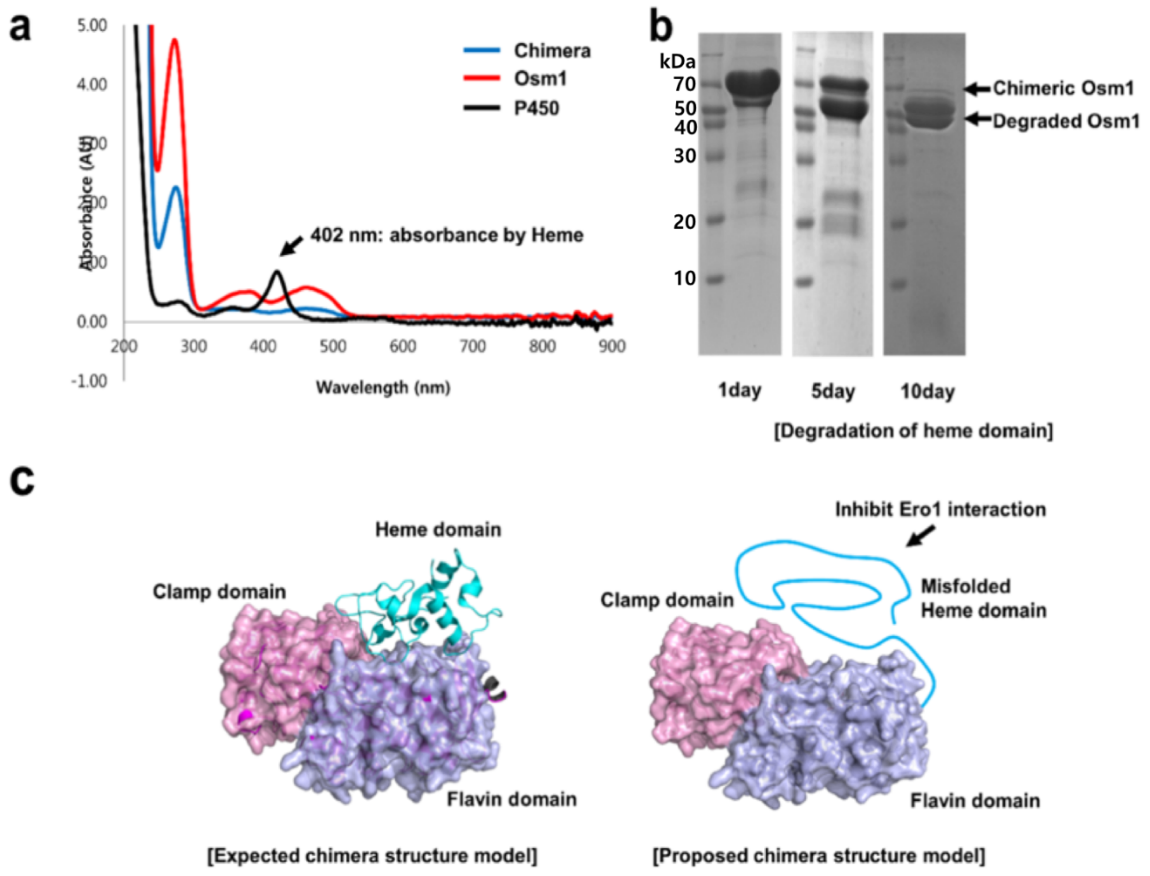

Supplementary Figure 8

Characterization of Chimeric Osm1 containing a heme domain. a Absorbance scan of Osm1 chimera to detect Heme. P450 containing Heme in the protein was used as positive control. Osm1 that does not contain Heme was used as negative control. Chimera did not exhibit a peak at 402 nm (blue line) indicated that Chimera do not contain Heme in the fused Heme domain. b Osm1 chimera proteins were subjected to SDS-PAGE and degradation patterns in the time scale were analyzed. c Proposed chimera structure model. Misfolded fused heme domain still inhibit the Ero1 interaction.

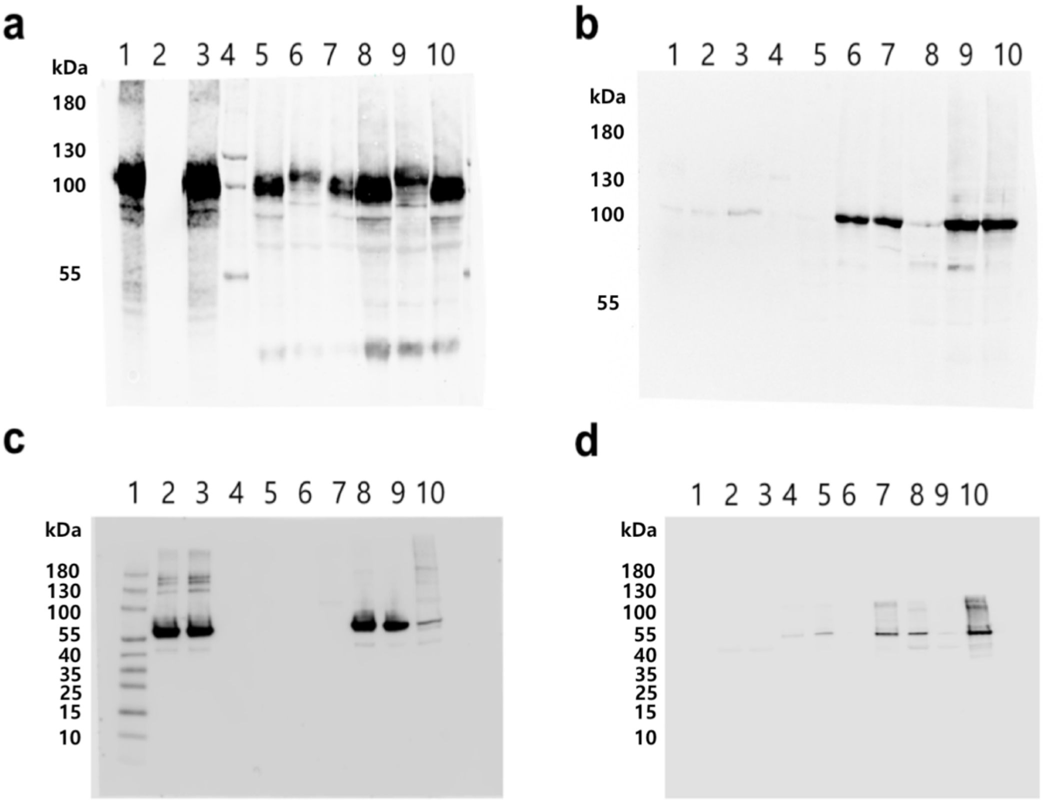

Supplementary Figure 9

Uncropped scans of the blots. a and b Uncropped gel for figure 4b. Lane1: Flag IP sample – Osm1-deletion + Ero1-flag. Lane2: Flag IP sample – Osm1-HA + Ero1-3xMyc. Lane3: Flag IP sample – Osm1-HA + Ero1-flag. Lane4: size marker. Lane5: 5% input – Osm1-deletion + Ero1-flag. Lane6: 5% input – Osm1-HA + Ero1-3xMyc. Lane7: 5% input – Osm1-HA +Ero1-flag. Lane8: 10% input – Osm1-deletion + Ero1-flag. Lane9: 10% input – Osm1-HA + Ero1-3xMyc. Lane10: 10% input – Osm1-HA +Ero1-flag. Lane 2,3/9,10 were used for figure 4b. c and d Uncropped gel for figure 4C. Lane1: size marker. Lane2~4: His-Osm1. Input (2), flow (3), and elution (4). Lane5~7: MBP-Ero1pc. Input (5), flow (6), and elution (7). Lane8~10: MBP-Ero1pc/Osm1 co-expression. Input (8), flow (9), and elution (10). Lane 2,3,4 / 8,9,10 were used for figure 4c.

Supplementary Tables

Supplementary Table1. Data collection and refinement statistics

	Osm1	2nd FAD-bound Osm1
**Data collection**		
Space group	$P2_1$	$P2_1$
Cell dimensions		
a, *b*, *c* (Å)	43.63, 109.27, 49.93	43.88, 109.32, 49.93
α, β, γ (°)	90.00, 112.55, 90.00	90.00, 112.24, 90.00
Resolution (Å)	50-1.80 (1.84-1.80) *	50-1.75 (1.79-1.75) *
R_{sym}	5.9% (25.7%)	6.1% (29.6%)
$I / \sigma I$	37.0 (5.8)	34.2 (5.4)
Completeness (%)	99.7 (100)	94.5 (100)
Redundancy	4.3 (4.3)	4.0 (4.2)
**Refinement**		
Resolution (Å)	28.6-1.8	26.5-1.75
No. reflections	36,796	41,288
R_{work} / R_{free}	17.9%/21.3%	18.8%/23.9%
No. atoms	3,788	3,989
Protein	3,617	3,616
Ligand/ion	61	92
Water	110	281
B-factors		
Protein	25.8	27.7
Ligand/ion		
Fumarate	29.3	28.0
FAD	25.2	27.6
2nd FAD (FMN)		35.4
Water	19.5	20.9
R.m.s. deviations		
Bond lengths (Å)	0.007	0.007
Bond angles (°)	1.157	1.135

*Values in parentheses are for highest-resolution shell.

[#]One crystal was used for 360 images data collection oscillated by 1°

Supplementary Table 2. Structural similarity search using DALI

**Proteins and accession numbers**	**Z-score**	**RMSD (Å)**	**Identity (%)**
Flavocytochrome C3 (Fcc3) from Shewanella frigidimarina (1QJD)	51.7	1.6	32
Fcc3 of shewanella putrefaciens strain mr-1 (1D4C)	46.8	1.9	33
Open form of Fcc3 from Shewanella frigidimarina (1QO8)	46.6	4.0	32
3-ketosteroid dehydrogenase from Rhodococcus jostii (4AT2)	40.2	4.3	23
Fumarate oxidoreductase from Escherichia coli (3P4S)	37.6	2.3	28

Supplementary Table 3. Catalytic activities of Osm1 with free Flavin molecules

	Vmax for free flavin oxidation (μM/sec)	Km (μM)	Vmax/Km (1/sec)
Osm1 + FAD	2.56e-04 (±2.579e-05)	2.858 (±0.903)	0.09
Osm1 + FMN	2.26E-04 (±3.09E-05)	5.847 (±2.0065)	0.039
Osm1 + Riboflavin	2.68E-04 (±6.67E-05)	9.3745 (±4.991)	0.029
S78K/P162R + FAD	2.08E-4	42.55	0.0049
